# Real-time tilting and twisting motions of ligand-bound states of α7 nicotinic acetylcholine receptor

**DOI:** 10.1007/s00249-023-01693-6

**Published:** 2024-01-17

**Authors:** Yue Yang, Tatsuya Arai, Daisuke Sasaki, Masahiro Kuramochi, Hidetoshi Inagaki, Sumiko Ohashi, Hiroshi Sekiguchi, Kazuhiro Mio, Tai Kubo, Yuji C. Sasaki

**Affiliations:** 1 Graduate School of Frontier Sciences, The University of Tokyo, Kashiwa, 277-8561 Japan; 2 AIST-UTokyo Advanced Operando-Measurement Technology Open Innovation Laboratory (OPERANDO-OIL), National Institute of Advanced Industrial Science and Technology (AIST), Kashiwa, 277-8565 Japan; 3 Graduate School of Science and Engineering, Ibaraki University, Hitachi, 316-8511 Japan; 4 Biomedical Research Insitute, National Institute of Advanced Industrial Science and Technology (AIST), Tsukuba, 305-8566 Japan; 5 Center for Synchrotron Radiation Research, Japan Synchrotron Radiation Research Institute, 1-1-1, Kouto, Sayo-cho, Sayo-gun, Hyogo 679-5198 Japan

**Keywords:** nAChR α7, Ivermectin, X-ray single molecule, Dynamics, Rotational motions

## Abstract

The α7 nicotinic acetylcholine receptor is a member of the nicotinic acetylcholine receptor family and is composed of five α7 subunits arranged symmetrically around a central pore. It is localized in the central nervous system and immune cells and could be a target for treating Alzheimer’s disease and schizophrenia. Acetylcholine is a ligand that opens the channel, although prolonged application rapidly decreases the response. Ivermectin was reported as one of the positive allosteric modulators, since the binding of Ivermectin to the channel enhances acetylcholine-evoked α7 currents. One research has suggested that tilting motions of the nicotinic acetylcholine receptor are responsible for channel opening and activation. To verify this hypothesis applies to α7 nicotinic acetylcholine receptor, we utilized a diffracted X-ray tracking method to monitor the stable twisting and tilting motion of nAChR α7 without a ligand, with acetylcholine, with Ivermectin, and with both of them. The results show that the α7 nicotinic acetylcholine receptor twists counterclockwise with the channel transiently opening, transitioning to a desensitized state in the presence of acetylcholine and clockwise without the channel opening in the presence of Ivermectin. We propose that the conformational transition of ACh-bound nAChR α7 may be due to the collective twisting of the five α7 subunits, resulting in the compression and movement, either downward or upward, of one or more subunits, thus manifesting tilting motions. These tilting motions possibly represent the transition from the resting state to channel opening and potentially to the desensitized state.

**Supplementary Information:**

The online version contains supplementary material available at 10.1007/s00249-023-01693-6.

## Introduction

Ion channels are directly and/or indirectly involved in ion flux through the cell membrane, which leads to changes in cell excitability. Ion flux triggers subsequent signaling cascades in the cell and to the adjacent cells and contributes to homeostasis. The nicotinic acetylcholine receptor (nAChR) represents a ligand-gated ion channel (LGIC) that alters the ionic permeability of the membrane by binding acetylcholine or cholinergic ligands. Because of the high density of the receptor in the electroplax of Torpedo and the advancement of cDNA cloning technologies, biochemical and structure–function studies on the nAChR in the neuromuscular junction (NMJ) have accumulated (Weill et al. 1974; Noda et al. 1983) and have extended to the nAChRs in the central and peripheral nervous system (Albuquerque et al. 2009)and the immune system (Fujii et al. 2017). The nAChR in the NMJ consists of four kinds of homologous subunits assembled in a molar stoichiometry of α2, β, γ(ε), and δ, and the α-subunit harbors the ACh-binding site (Karlin et al. 1971; Klymkowsky and Stroud 1979; Unwin 2014). The binding of agonistic ligands to nAChR induces ion flux through the channel formed by the pentameric subunits. Patch-clamp technology enabled insight into the electrophysiological behavior of a single channel of the nAChR (Sakmann and Neher 1984), and together with kinetic studies, it was assumed that the nAChR has at least three different molecular dynamic substates in thermal reversible equilibrium: a basal or resting state (R), an active open channel state (A) and at least one desensitized state (D) (Changeux and Edelstein 1998). Several approaches, such as X-ray crystallography, electron/cryo-electron microscopy (Nemecz et al. 2016; Rahman et al. 2020), and in silico model analysis (Taly et al. 2005), can be used to visualize these states, which are possibly accompanied by conformational changes. However, there are currently no precise structural data providing details of the physiological transition in the receptor itself.

Moreover, Nigel Unwin’s gating movement model (Unwin and Fujiyoshi 2012) suggests that the binding of ACh to the α subunits of the nAChR may cause a conformational change in the protein structure. Specifically, the inner β-sheet of α subunits of nAChR may push out their adjacent neighbors, resulting in a tilting motion. This tilting motion is thought to open the channel pore, allowing the flow of ions across the cell membrane.

Neuronal nAChR α7 consists of homopentamer subunits arranged symmetrically around a central pore. It is highly expressed in both the hippocampus and cerebral cortex, suggesting the involvement of α7 in higher-order neural functions such as learning and memory (Dani and Bertrand 2007). Furthermore, significant correlations were shown between the impairment of α7 signaling and the cognitive deficits associated with Alzheimer’s disease and schizophrenia (Freedman et al. 1995, 1997; Qi et al. 2007). For a therapeutic drug to treat Alzheimer's disease and cognitive impairment associated with schizophrenia (CIAS), α7 signaling in the CNS would need to be augmented without affecting ACh binding to the allosteric site(s), which leads to favorable conformational change. Ivermectin (IVM) has been classified as a type I positive allosteric modulator (PAM) for the α7 nAChR, and has been found to transiently potentiate α7 activity at micromolar concentrations when administered before ACh. Interestingly, α7 does not exhibit any direct response to IVM, as reported in previous studies (Krause et al. 1998).

**Fig. 1:**
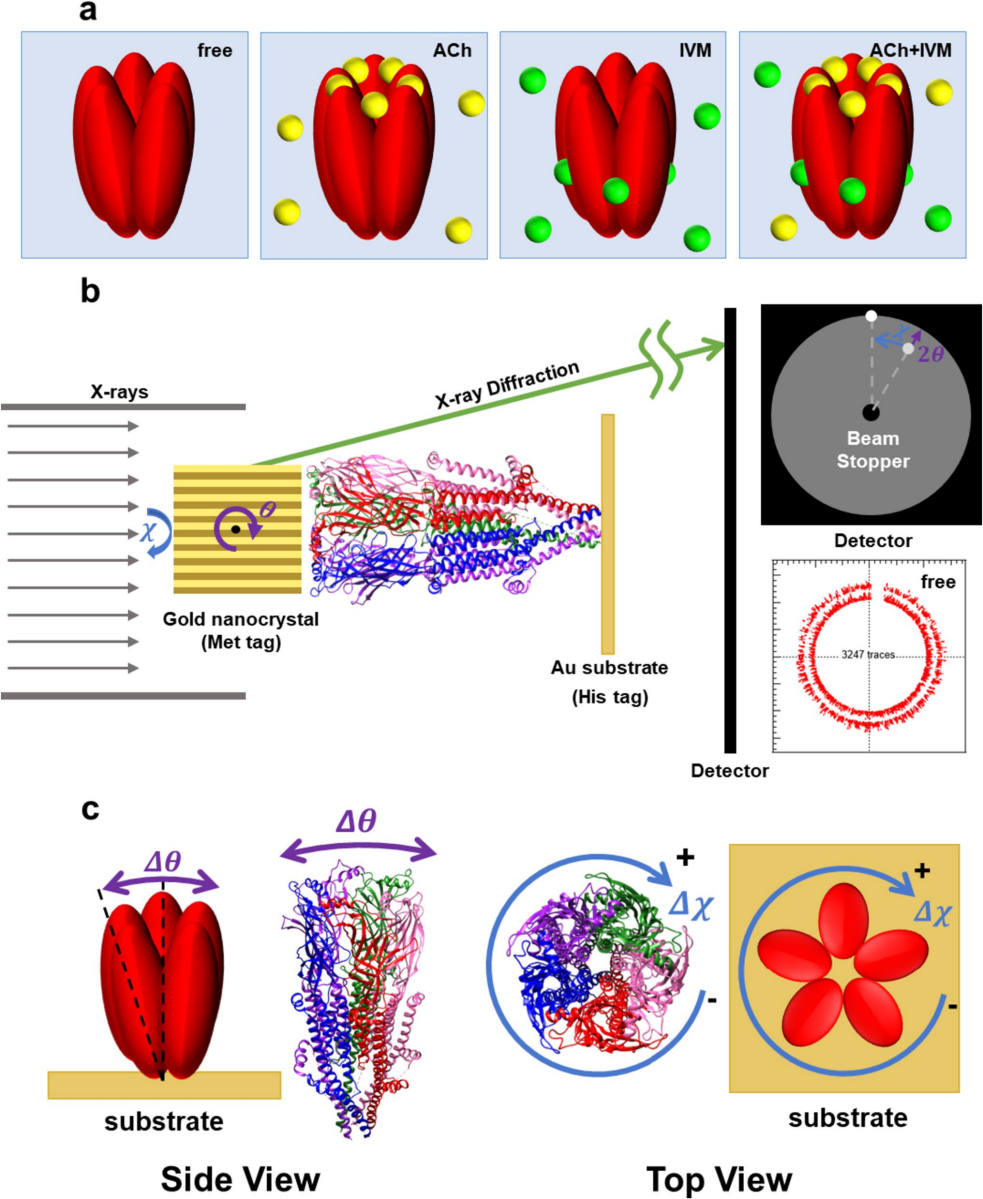
Schematic diagram of experiments. **a** Schematic diagram of four experimental conditions: without ligand, with ACh (yellow rounds represent the ACh molecules), with IVM (green rounds represent the IVM molecules), and with both ACh and IVM (ACh + IVM). **b** Schematic diagram of nAChR α7 with tilting (θ) and twisting (χ) motions. nAChR α7 was labeled with gold nanocrystals via the Met tags and fixed to the gold substrate by His tags to measure the twisting (χ) and tilting (θ) motions, not the translation motion (Fujii et al. 2017). **c** Schematic illustration of two modes of movement of nAChR α7 measured by the DXT method. By using the DXT method, the rotational motions of nAChR α7 could be monitored by tracking the X-ray diffraction spots from labeled gold nanocrystals

Here, to test whether Unwin’s gating movement model applies to nAChR α7 and acquire more precise information on the 3D molecular motions of nAChR α7, we applied the diffracted X-ray tracking (DXT) method to investigate the dynamic behaviors of nAChR α7 under various conditions, including the ligand-free state (resting state), the opening channel state and possibly desensitized state by binding with agonistic ligand ACh, the closed state by binding with the type I positive allosteric modulator (PAM) IVM, and the state by binding with both of ACh and IVM (Fig. 1a). DXT is a method to track X-ray diffraction spots from gold nanocrystals labeled on an individual single protein in real time and real space (Sasaki et al. 2000). The positions where these rounds are located represent their actual binding positions on nAChR α7. nAChR α7 was labeled with gold nanocrystals (20–50 nm) via Met tags and fixed to the gold substrate by His tags. Due to the limited translational motions, we define the patterns and directions of twisting (χ) and tilting (θ) motions of nAChR α7, as shown in Fig. 1b, c.

## Method

### Diffracted X-ray tracking

We performed diffracted X-ray tracking in the Spring-8 BL40XU beamline by using continuous white X-ray illumination to monitor the twisting (χ) and tilting (θ) motions of nAChR α7 without a ligand, with ACh, with IVM, and with both ACh and IVM (ACh + IVM), with a time-resolution of 100 μs. The time window was chosen based on previous research where the internal motion differences of nAChR and transient receptor potential vanilloid type 1 protein were successfully measured over 100-μs time intervals (Sekiguchi et al. 2014; Mio et al. 2023) in both the tilting (θ) and twisting (χ) directions. Moreover, millisecond-level resolution is not enough for monitoring protein rotational motion, and even microsecond-level resolution may not immediately monitor protein rotational motion. We conducted 50 sets of repeated experiments, each consisting of 500 frames, for each ligand condition. Each receptor sample is used for one ligand condition due to the multiple exposures to radiation damage and the possibility of detachment of α7AChR from the substrate. nAChR α7 was labeled with gold nanocrystals (20–50 nm) via Met tags and fixed to the gold substrate by His tags. Although gold nanocrystals are much larger than receptors, previous experiment results (Fig. S1 in the supplemental information online) demonstrate that the size of gold nanocrystals only affects the intensity of the diffraction spot and has no significant effect on the motion of α7 nAChR. Also, before this research, we conducted DXT experiments with different tag positions and obtained consistent results (Baba et al. 2017). The X-ray diffraction spots in the Au(111) and Au(200) regions were recorded by an X-ray image intensifier and a CMOS camera, as shown in Supplementary Video S1. More detailed parameters of the DXT experiments can be found in the Supplemental Information Online Table S1.

### DXT sample preparation

The cDNA encoding rat α7 nicotinic acetylcholine receptor (GeneBank S53987) was cloned by PCR using the newborn rat brain cDNA library as a template. We expressed the rat nAChR α7 (UniProt Q05941, Gly1-Ala480) in Xenopus oocytes by co-expressing the nAChR chaperones ric3 and NACHO, and the receptor was purified by the reported procedures (Bergeron et al. 2011). To let nAChR α7 both connect to gold nanocrystals and gold substrates in the DXT experiment, we inserted a Met tag (MGGMGGM) (Sekiguchi et al. 2014) between the Pro17 and Leu18 in the N-terminal extracellular region, and a His tag between the Arg322 and Met323 in the cytoplasmic loop region. We confirmed that the α7 construct with the Met and His tags exerts ACh-induced currents in Xenopus oocytes.

In the DXT experiments, for gold-substrate preparation, we diluted 1 mg/ml of dithiobis (C2-NTA) to 0.8 mM with anhydrous ethanol and immersed the gold substrates into dithiobis solution for 24 h and then in a 100 mM NiSO_4_ solution for 24 h.

After the gold substrates were washed with phosphate-buffered saline (PBS, pH7.4), each substrate was dripped with 18 μl of nAChR α7 solution and incubated at 4 °C for 6 h. The proteins were fixed to the gold substrate by forming complexes of His residues and Ni–NTA. Each gold substrate was dripped with 60 μl of gold nanocrystal solution and incubated for 2 h.

To prepare for the experiment, the unbound gold substrates were first washed with PBS. The gold substrate was dripped with 6 μl of PBS solution, 100 μM ACh or 30 μM IVM, for the ligand-free, ACh, or IVE condition, respectively. For the ACh + IVM condition, we preexposed nAChR α7 in 3 μl of 30 μM IVM solution for 1 min, and then we added 3 μl of 200 μM ACh-PBS solution to the gold substrate.

### Angular displacement analysis

**Fig. 2:**
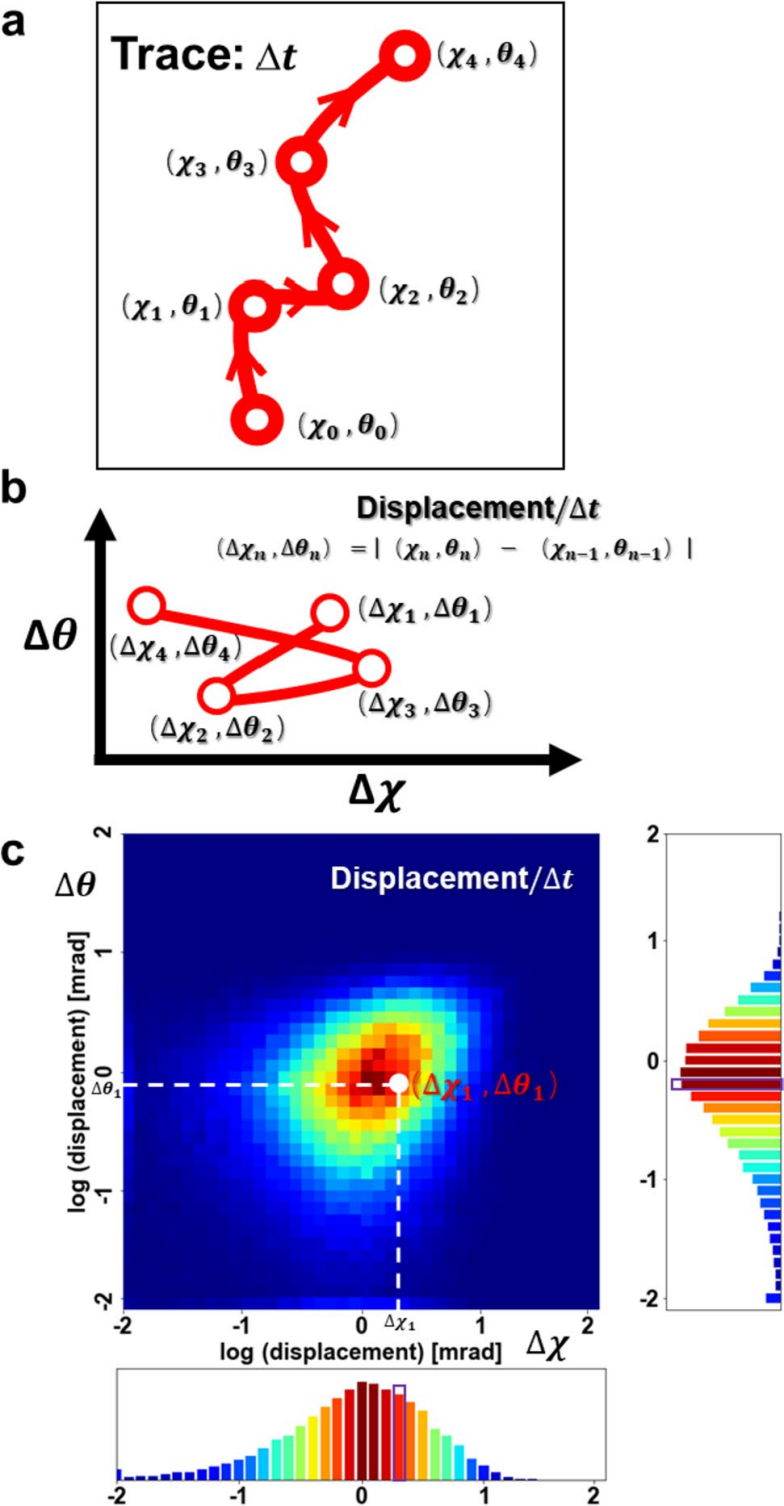
Schematic diagram of angular displacement analysis. **a** Schematic diagram of the positions of a series of diffraction spots changed over unit time interval $\Delta t$, from (${\chi }_{0},{\theta }_{0}$) to (${\chi }_{1},{\theta }_{1}$), (${\chi }_{2},{\theta }_{2}$), (${\chi }_{3},{\theta }_{3}$) and so on. **b** Schematic diagram of the absolute value of angular displacement changed with the time intervals $\Delta T$. **c** Schematic diagram of a normalized 2D motion map as a time interval of $\Delta t$. The color of the white spot here represents the probability of the sample’s angular displacement is (${\Delta \chi }_{1},\Delta {\theta }_{1}$)

Through the diffraction images obtained by DXT method, the most important information is the positions of a series of diffraction spots changed over time. We combined them and called as trajectories, as shown in Fig. 2a. The initial diffraction spot is located at (${\chi }_{0},{\theta }_{0}$) and changed the position over time by a unit time interval $\Delta t$ to (${\chi }_{1},{\theta }_{1}$), (${\chi }_{2},{\theta }_{2}$), (${\chi }_{3},{\theta }_{3}$) and so on. Based on this, the absolute value of angular displacement that changes with different time intervals $\Delta T$ can be calculated. Assume that the time intervals $\Delta T$ exactly is a unit time interval $\Delta t$, then the formula of angular displacement becomes:

$$\left( {\Delta \chi_n ,\Delta \theta_n } \right) = \left| {\left( {\chi_n ,\theta_n } \right) - \left( {\chi_{n - 1} ,\theta_{n - 1} } \right)} \right|\quad n = 1,2,3 \ldots$$

If the time intervals $\Delta T$ is equal to $2\Delta t$, the angular displacement becomes:

$$\left( {\Delta \chi_n ,\Delta \theta_n } \right) = \left| {\left( {\chi_{2n} ,\theta_{2n} } \right) - \left( {\chi_{2n - 2} ,\theta_{2n - 2} } \right)} \right|\quad n = 1,2,3 \ldots$$

By extension, if the time intervals $\Delta T$ is equal to m times $\Delta t$, then the angular displacement becomes:

$$\left( {\Delta \chi_n ,\Delta \theta_n } \right) = \left| {\left( {\chi_{mn} ,\theta_{mn} } \right) - \left( {\chi_{mn - m} ,\theta_{mn - m} } \right)} \right|\quad n = 1,2,3 \ldots$$

It is easy to see that the magnitude and the trend of the absolute value of angular displacement will change with the time intervals $\Delta T$ (Fig. 2b). The angular displacement curve of a single diffracted spot can only reflect its own motion mode but cannot provide the overall motion mode information of whole protein. In contrast, the distribution of angular displacements in the twisting and tilting motion directions, obtained by calculating the trajectories of all diffracted spots under different conditions (free: 3247 diffraction spots; ACh: 2020 diffraction spots; IVM: 2157 diffraction spots; ACh + IVM: 1244 diffraction spots), is considered to have the function of reflecting the overall motion pattern. To determine the complete 3D rotational motion of nAChR α7 more comprehensively and accurately, we combined the angular displacement distribution of gold nanocrystals in both the χ and θ directions as a normalized 2D motion map (Sekiguchi et al. 2014; Oishi et al. 2023) as a time interval of $\Delta t$ (Fig. 2c). The color in the 2D motion represents the probability of the absolute value of the angular displacement belonging to a small range of motions. The closer the color is to red, the greater probability of the twisting and tilting motion corresponding to that point.

## Results and discussion

**Fig. 3:**
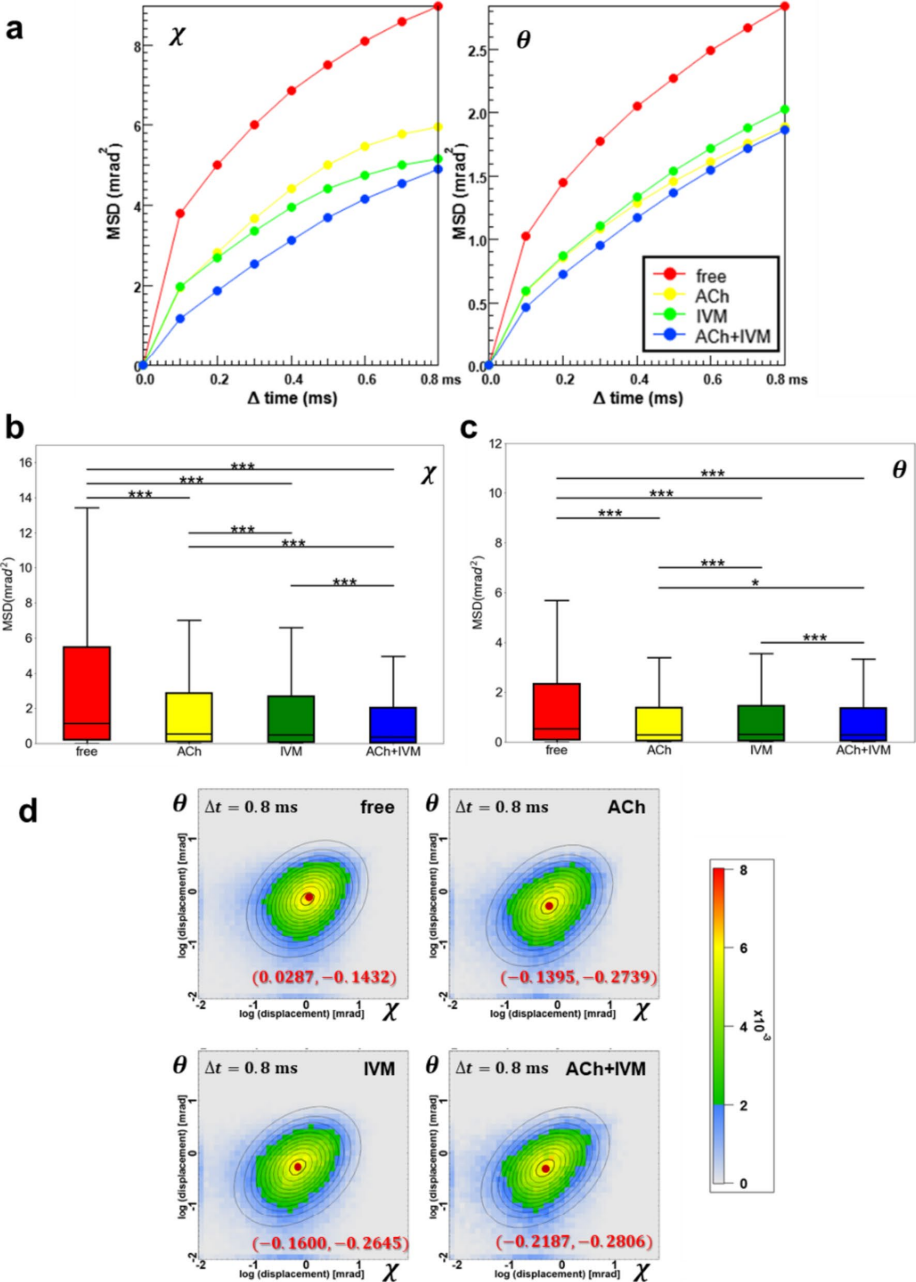
DXT analyses. **a** MSD curves of nAChR α7 in the twisting and tilting motion in the presence of nothing (red), ACh (yellow), IVM (green), or ACh + IVM (blue), showing the order of the degree of motion of nAChR α7 in 0.8 ms. The boxplot analysis (displaying median values of MSD) and Wilcoxon Rank-Sum Test of MSD values for nAChR α7 in the **b** twisting and **c** tilting directions at a time interval of 0.8 ms at four conditions. Asterisk shows that *p*-value between different conditions is statistically significant (*p < 0.1, **p < 0.01, ***p < 0.001). **d** Normalized 2D motion maps of nAChR α7 in ligand-free condition and in the presence of ACh, IVM, or ACh + IVM. All angular displacements are presented as absolute values. The 2D Gaussian distribution center of the displacement of nAChR α7 at four different ligand conditions, representing their main motion modes

**Fig. 4:**
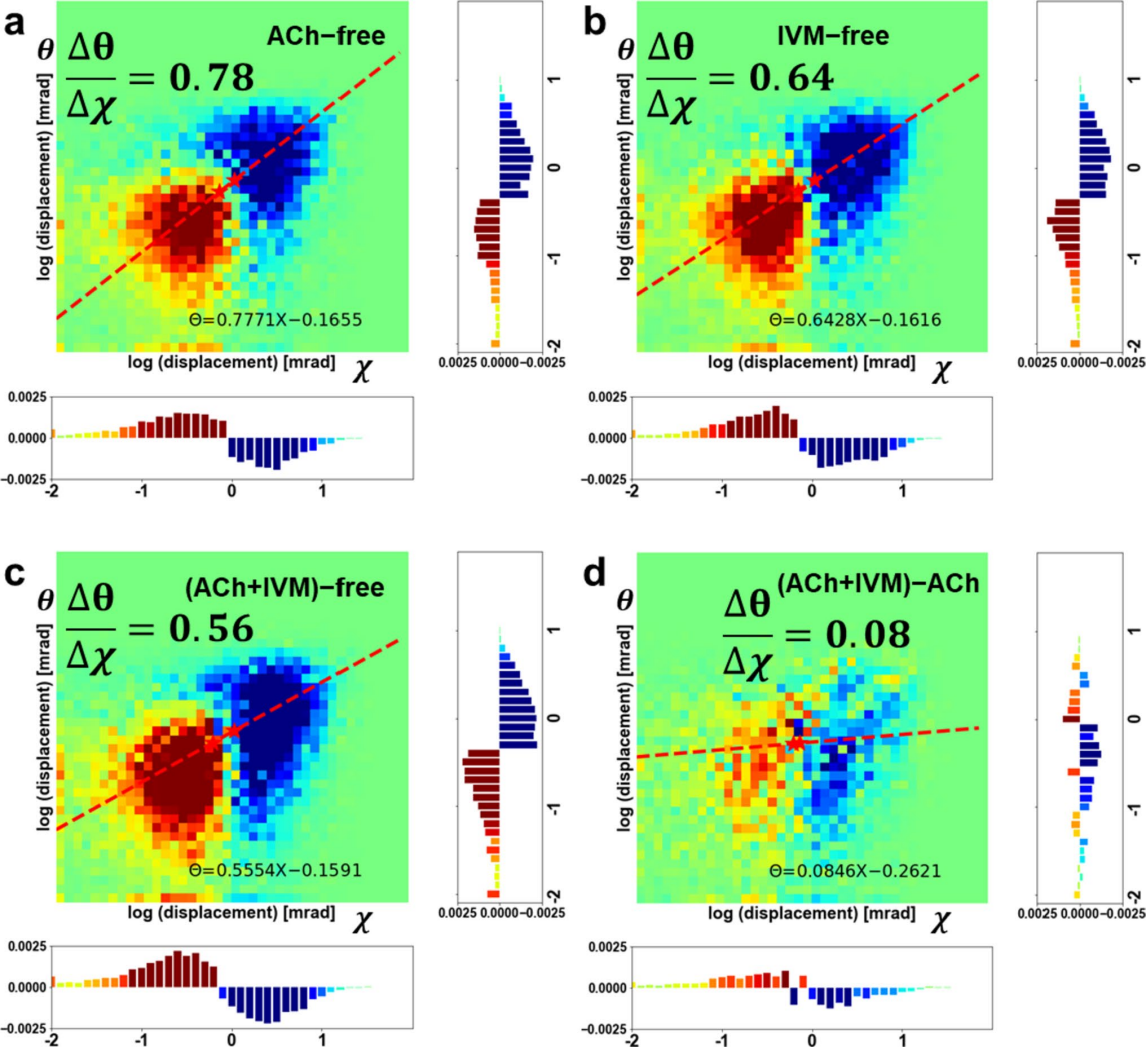
Difference 2D motion maps. Difference 2D motion maps between two conditions between each of the three ligands with free condition (**a**–**c**), and between ACh + IVM and ACh (**d**). The two red pentagrams represent the main motion mode of two conditions, and the red dashed line represents the line connecting these two center points. These results show that the combination of nAChR α7 with the ligands affects its overall rotational motion and the difference between ACh + IVM and ACh almost entirely depends on the twisting (χ) motion

Mean-square-displacement (MSD) analysis is often used in single-molecule tracking (Saxton and Jacobson 1997) to determine the degree of molecular motion and is also used in the analysis of DXT data (Shimizu et al. 2008; Sekiguchi et al. 2013, 2014). Figure 3a shows the MSD curves derived from the angular-displacement distribution of the gold nanocrystals under four experimental conditions: ligand-free, in the presence of ACh, in the presence of IVM, and in the presence of both ACh and IVM (ACh + IVM). For the twisting (χ) direction, the degree of motion of nAChR α7 in order from largest to smallest was ligand-free, ACh, IVM and ACh + IVM. For the tilting (θ) direction, the degree of motion of nAChR α7 in order from largest to smallest was ligand-free, IVM, ACh and ACh + IVM. Both results show that bound ligands could restrict the freedom of nAChR α7 and decrease the twisting (χ) and tilting (θ) motions. Also, the boxplot analysis (displaying median values of MSD) and Wilcoxon Rank-Sum Test of MSD values (using a 0.8 ms time interval) for 4 conditions (Fig. 3b for χ motions and Fig. 3c for θ motions) indicate that there was a statistically significant mean difference in the distribution of MSD values among the four conditions in both twisting and tilting direction. Then, in alignment with the previous research (Sekiguchi et al. 2014)which successfully measured motions of nAChR without damaging the sample, we selected 0.8 ms as a time interval $\Delta t$ and plotted 2D motion maps for four different conditions (Fig. 3d). Additionally, to confirm that our samples in the experiment were not damaged by the X-ray radiation, we also calculated the correlation between the MSD curves of the free condition from the first 25 times DXT experiments and the last 25 times DXT experiments. As shown in Fig. S2, it is evident that the nAChR α7 were not compromised. By fitting a 2D Gaussian distribution, we determined the center peak of the displacement of nAChR α7 at four different ligand conditions, representing the main motion modes. The results are the same as the MSD values. The magnitudes of both twisting and tilting motions in the free condition were significantly bigger than those in the other conditions, while the tilting motions did not differ much among the three ligands-bound conditions, and their main differences are in the twisting direction. Furthermore, due to the previous electrophysiological results, it is known that nAChR α7 can be activated by ACh to open the ion channel; however, prolonged application results in a decrease in the response, which means desensitization. We consider the ACh condition to be in a nearly desensitized state, as the sample adjustment time and measurement time are approximately one minute. The ACh-evoked α7 currents can be enhanced by IVM-preapplication (Krause et al. 1998); however, the ion channel cannot open only in the presence of IVM. Thus, we classified the three ligand conditions into two groups based on whether the ion channel of nAChR α7 can be opened. We are interested in understanding the effect of different ligands on the dynamics of nAChR α7, especially in exploring the dynamics changed by IVM. Furthermore, as shown in Fig. 3b, c, the independence of sample motion under these four conditions has already been demonstrated. Therefore, we produced the difference 2D motion maps between each of the three ligands with free condition (Fig. 4a–c), and show the differences between ACh + IVM and ACh (Fig. 4d), ACh and IVM (Fig. S3a in the supplemental information online), ACh + IVM and IVM condition (Fig. S3b in the supplemental information online). Taking the difference 2D motion map between ACh and free condition as an example (Fig. 4a), the two red pentagrams represent the center points obtained from the Gaussian fits of two conditions in Fig. 3d, and the red dashed line represents the line connecting these two center points. The significance of the red dashed line is shown in Fig. S3c, d. When it is aligned horizontally with the χ axis, it indicates that the main difference between the two different conditions lies in the twisting motion. When it is aligned horizontally with the θ axis, it indicates that the main difference between the two different conditions lies in the tilting motion. It is easy to see that, compared to the three ligand conditions, the results of the free condition cannot be specified to be related to only one direction of motion, suggesting that the combination of nAChR α7 with the ligands affects its overall rotational motion. The results of the motion difference between ACh and IVM are the same as Fig. 3a. On the other hand, Fig. 4d highlights that the difference between ACh + IVM and ACh almost entirely depends on the twisting (χ) motion. This indicates that the twisting (χ) motions of nAChR α7 were inhibited in the presence of IVM; however, the tilting (θ) motions did not change much. Similarly, Fig. S3b showed that the twisting (χ) and tilting (θ) motions of nAChR α7 were inhibited in ACh's presence.

**Fig. 5:**
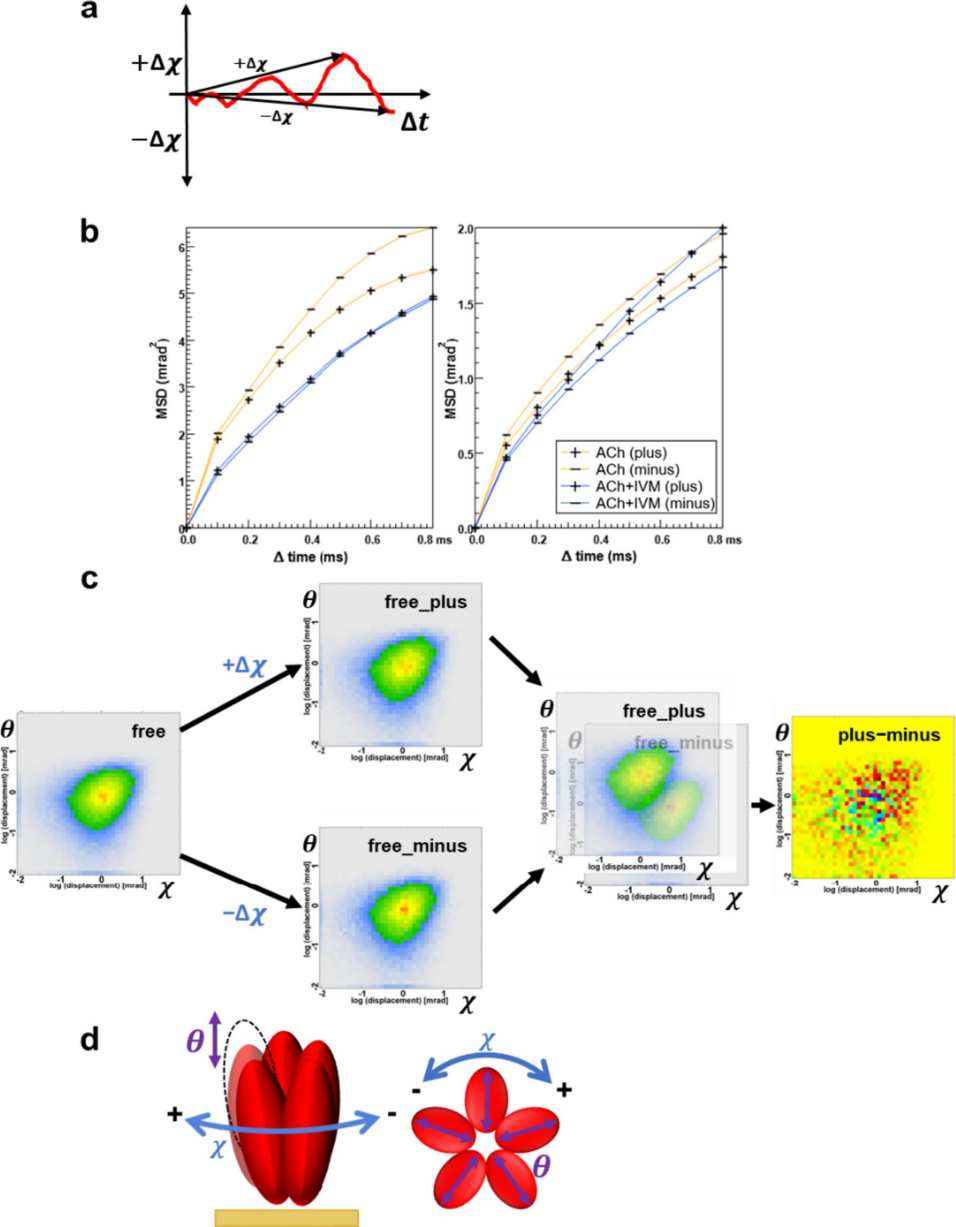
Difference 2D motion maps between the positive and negative directions in the χ axis. **a** Schematic diagram of the positive and negative directions in the χ axis at a time interval $\Delta t$. **b** MSD curves of twisting and tilting motions of nAChR α7 in the positive and negative directions of the χ axis in the presence of ACh or ACh_IVM, showing that IVM can twist nAChR α7 in the direction opposite to what it is when activated by ACh. **c** Schematic diagram of how to produce the difference 2D motion maps between the positive and negative directions in the χ axis for all conditions. **d** Schematic diagram of the ion channel opening of nAChR α7. Five α7 subunits are twisted together, and one or more of them are squeezed and moved downward or upward

Moreover, because the displacements shown in the 2D motion maps were absolute values here and the changes of different conditions in the χ axis are more significant, we separated the overall motions according to the positive and negative directions in the χ axis at a time interval $\Delta t$ (Fig. 5a). We defined that the positive direction of the twisting motions is clockwise (CW) order, and the negative direction is counterclockwise (CCW) order. However, for the θ axis, because only the top and bottom of the protein are fixed, the positive and negative directions of the θ axis are meaningless, only the magnitude is meaningful. At this point, the 2D motion map for each condition can be divided into two images along the positive and negative directions in the χ axis with time intervals of 0.8 ms (Fig. S4 in the supplemental information online). The MSD curves in Fig. 5b (complete version in Fig. S5a, supplemental information online) indicated that the degree of motion of nAChR α7 ordered from largest to smallest was ACh (χ mode along negative direction: negative direction), ACh (χ mode along positive direction: positive direction), ACh + IVM (positive direction) and ACh + IVM (negative direction). This result indicates that the twisting (χ) motions observed in ACh and ACh + IVM conditions are opposite to each other, which demonstrates that IVM can twist nAChR α7 in the direction opposite to that of the ACh-bound possible desensitized state, may indicate that the mechanism of nAChR α7 channel opening is related to the different twisting (χ) directions and degrees of movements.

To analyze the twisting and tilting motion modes of the positive and negative directions of the χ axis, we used a normal Gaussian distribution to fit the histogram of the angular-displacement distribution of each condition (Fig. S6 in the supplemental information online). From that, we were most interested in the difference between the ACh and IVM condition. Using the peak position, it can be roughly judged that in the presence of IVM, nAChR α7 is inclined to move in the positive direction (twist in CW order), and in the presence of ACh, nAChR α7 is inclined to move in the negative direction (twist in CCW order). In the θ direction (Fig. S6b), it was interesting that only the ACh-bound state of nAChR α7 tilted more when it twisted CCW. Also, the boxplot analysis (displaying median values of MSD) and Wilcoxon Rank-Sum Test of MSD values between the positive and negative directions along the χ axis for all conditions (Fig. S5b, c in the supplemental information online) reveal a statistically significant mean difference in the distribution of MSD values among the positive and negative directions for free, ACh, and IVM in the twisting direction. Unfortunately, the results for ACh + IVM in the twisting direction did not reach statistical significance. However, considering the unique opportunity, we also conducted the same analysis for ACh + IVM as we did for the other three conditions, and include the results in the supplemental information online. Therefore, to determine the specific motion patterns of each condition, we calculated the difference in the angular-displacement distribution in the positive and negative directions of the χ axis for each condition and used the difference in the Gaussian fitting results in the positive and negative directions in Fig. S7 as a simple fit. As shown in Fig. S7a, in the χ direction, the ligand-free nAChR α7 showed predominantly CW twisting. In the presence of ACh, it twisted more in the CCW order with channel opening and possibly desensitized, and in the presence of IVM, nAChR α7 twisted more in the CW order without channel opening. It was demonstrated that IVM can twist nAChR α7 CW; however, the ion channel opens and possibly undergoes desensitization only when nAChR α7 twists CCW, it does not open when nAChR is twisted CW. This result aligns with a previous cryo-EM study (Noviello et al. 2021), which found similar dynamics and highlighted the significance of intracellular and extracellular interactions involving the M2 helix in regulating these conformational changes. Specifically, upon activation of nAChR α7, the extracellular domain undergoes a counterclockwise twist, while during desensitization, this motion reverts to a more resting-like conformation, resembling a clockwise twist. Although the DXT method may not provide specific information about the conformational changes in nAChR α7 under different ligand-bound states, it still provides value in assessing the overall trends in dynamics. As shown in Fig. S7b, in the θ direction, the ligand-free nAChR α7 tilted more while it twisted CW. In the presence of ACh, it tilted more when it twisted CCW. In the presence of IVM, nAChR α7 tilted more when it twisted CW, similar to the ligand-free condition.

**Fig. 6:**
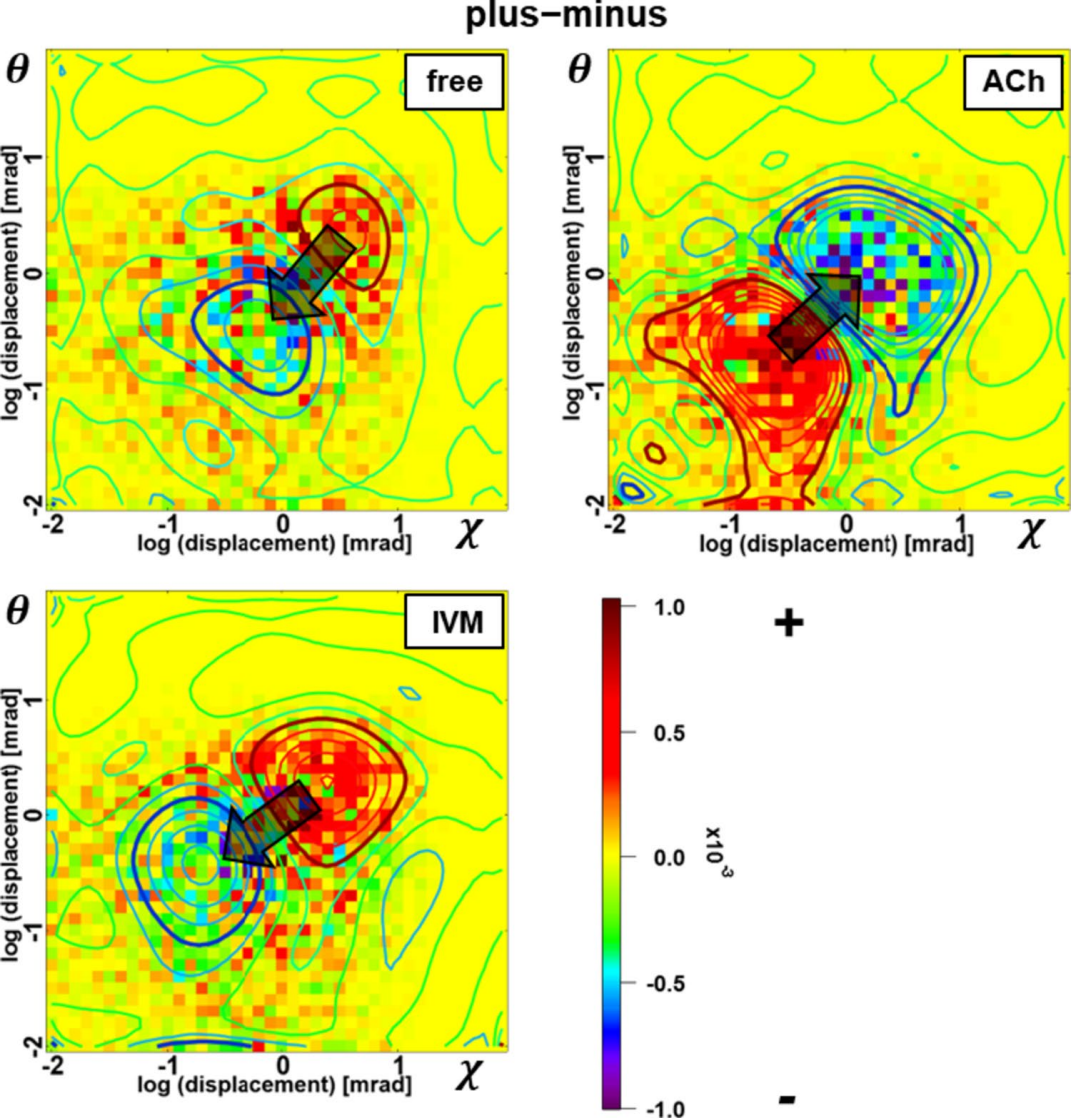
Difference 2D motion contour maps. The difference 2D motion contour maps between the positive and negative directions in the χ axis for free, ACh and IVM conditions. The red area is the area of positive χ motion, and the blue area is the area of negative χ motion

Consequently, we also generated difference 2D motion maps for all conditions (Fig. 6 and Fig. S8 in the supplemental information online), comparing the positive and negative directions along the χ axis. We used the 2D motion map obtained by summarizing the positive χ directional angular displacement data at a time interval of 0.8 ms in Fig. S4 and subtracted it from the negative χ directional angular displacement data to obtain the difference 2D motion map for conditions which are validated as independent (Fig. 5c). According to the contour plots fitted by the 2D polynomial (Fig. 6), we can see that in the ligand-free condition and the IVM condition, the 2D motion map can be divided into two areas, the area of positive motion in χ (red) and the area of negative motion in χ (blue). Those contour plots demonstrate that nAChR α7 showed positive twisting (χ) motions, while the tilting (θ) motions were stronger. However, in the presence of ACh, the 2D motion map could be divided into two areas but with inversive positions, meaning that nAChR α7 showed negative twisting (χ) motions and the tilting (θ) motions were stronger, opposite to the trends shown with IVM.

These findings suggest that when five α7 subunits are twisted together in CCW order within the ACh-bound α7 nAChR, one or more of them are squeezed and pushed upward by others, resulting in tilting movements (Fig. 5d). In contrast, in the presence of IVM, when five α7 subunits are twisted together in CW order, one or more of the subunits are squeezed in the opposite direction and pushed downward, also resulting in more tilting movements. These findings support Nigel Unwin’s gating movement model. The advantage of this study compared to previous research (Sekiguchi et al. 2014) is the discussion of more precise and stable positive and negative rotational motions. However, further understanding of the 3D dynamics of nAChR α7 is still required to confirm its validity. Additionally, due to the rapid desensitization of nAChR α7, it is challenging to clearly elucidate the twisting or tilting motions dependent on the differentiated number of subunits bound to ACh. To address this issue, real-time measurement of the motions just after exposure of ACh to the receptor is required. We are currently conducting experiments using Caged ACh (Nakamura et al. 2023) and employing the DXT method to analyze ligand concentration-dependent movements.

### Supplementary Information

Below is the link to the electronic supplementary material.

Supplementary file 1 (MP4 6480 KB)

Supplementary file 2 (DOCX 1891 KB)

## Data Availability

Derived data supporting the findings of this study are available from the corresponding author on request.
