# Supplementary material for: Real-time tilting and twisting motions of ligand-bound states of α7 nicotinic acetylcholine receptor
Source: Eur Biophys J. 2024 Jan 17;53(1-2):15–25. doi: 10.1007/s00249-023-01693-6 (PMC10853312; doi:10.1007/s00249-023-01693-6)
Supplement: Supplementary file 2 — (DOCX 1891 KB) [file 249_2023_1693_MOESM2_ESM.docx]

**Supplementary Information (SI) for European Biophysics Journal**

**Real-time tilting and twisting motions of ligand-bound states of α7 nicotinic acetylcholine receptor**

**Author information:**

**Authors:** Yue Yang^1^, Tatsuya Arai^1,2^, Daisuke Sasaki^1^, Masahiro Kuramochi^1,3^, Hidetoshi Inagaki^4^, Sumiko Ohashi^1,2^, Hiroshi Sekiguchi^5^, Kazuhiro Mio^2^, Tai Kubo^1,2^ and Yuji C. Sasaki^1,2,5*^

**Affiliations:**

^1^Graduate School of Frontier Sciences, The University of Tokyo, Kashiwa, 277-8561, Japan

^2^AIST-UTokyo Advanced Operando-Measurement Technology Open Innovation Laboratory (OPERANDO-OIL), National Institute of Advanced Industrial Science and Technology (AIST), Kashiwa, 277-8565, Japan

^3^Graduate School of Science and Engineering, Ibaraki University, Hitachi, 316-8511, Japan

^4^Biomedical Research Insitute, National Institute of Advanced Industrial Science and Technology (AIST), Tsukuba, 305-8566, Japan

^5^Center for Synchrotron Radiation Research, Japan Synchrotron Radiation Research Institute, 1-1-1, Kouto, Sayo-cho, Sayo-gun, Hyogo, 679-5198, Japan

*Corresponding author

Yuji C. Sasaki, Ph. D.

Email: [ycsasaki@edu.k.u-tokyo.ac.jp](mailto:ycsasaki@edu.k.u-tokyo.ac.jp)

**Contributions:** Y. Y., T. K., and Y.C.S. wrote the manuscript. Y. Y., T. K., T. A., and D. S. performed the experiments. Y. Y. and T. A. analyzed data.

**The results for ACh+IVM**

In the case of ACh+IVM, as observed in Fig. S7, it twisted more in the CCW order, but the twisting was not as strong as in the ACh condition, also with less tilting motion, suggesting a combination of the effects of ACh and IVM. Also, for the difference 2D motion maps between the positive and negative directions in the 𝝌 axis for ACh+IVM (Fig. S8), the situation was more complex. The 2D motion map could be divided into four areas, which seem to have two mode motions in each plus and minus direction, suggesting that may be the sum of the results from ACh and IVM conditions. Therefore, same as other conditions, we can say that the results are affected by both ACh and IVM, some α7 subunits may be squeezed and pushed upward and others move downward, resulting in less tilting; however, the ion channel still opens.

**Fig. S1 Preliminary experiment results**

**
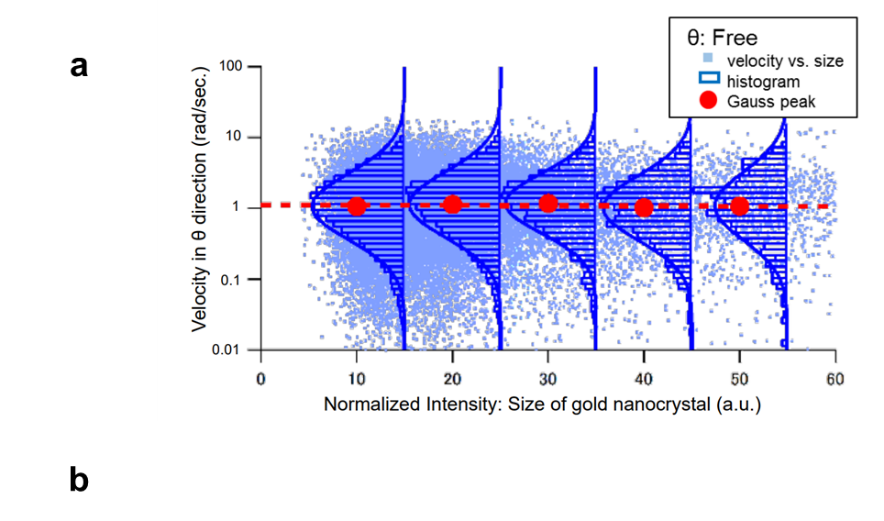
**

The relationship between the size of gold nanocrystal and the velocity in tilting direction of α7 nAChR. This result demonstrates that the size of gold nanocrystals only affects the intensity of the diffraction spot and has no significant effect on the motion of α7 nAChR.

**Fig. S2** **X-ray damage estimation
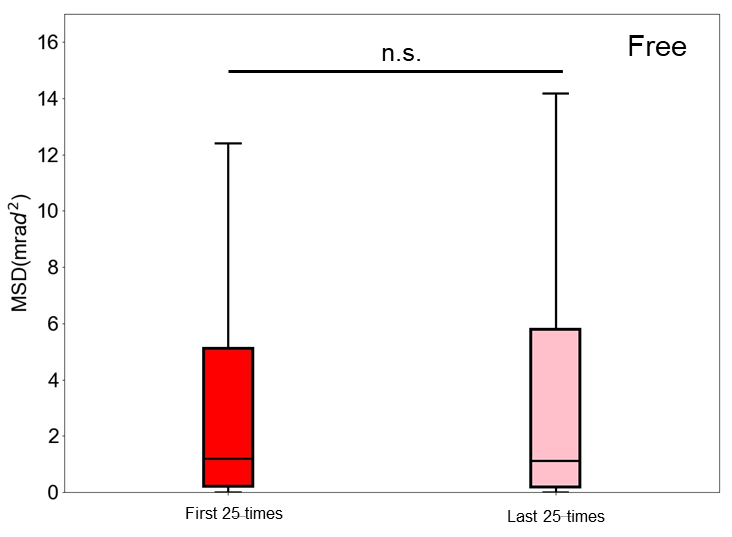
**

The boxplot analysis and Wilcoxon Rank-Sum Test of MSD curves of the free condition from the first 25 times DXT experiments and the last 25 times DXT experiments. Asterisk shows that p-value between two group is not statistically significant and suggest that nAChR α7 were not compromised by X-rays. (* p < 0.1, ** p < 0.01, *** p < 0.001).

**Fig. S3 Difference 2D motion maps**

**
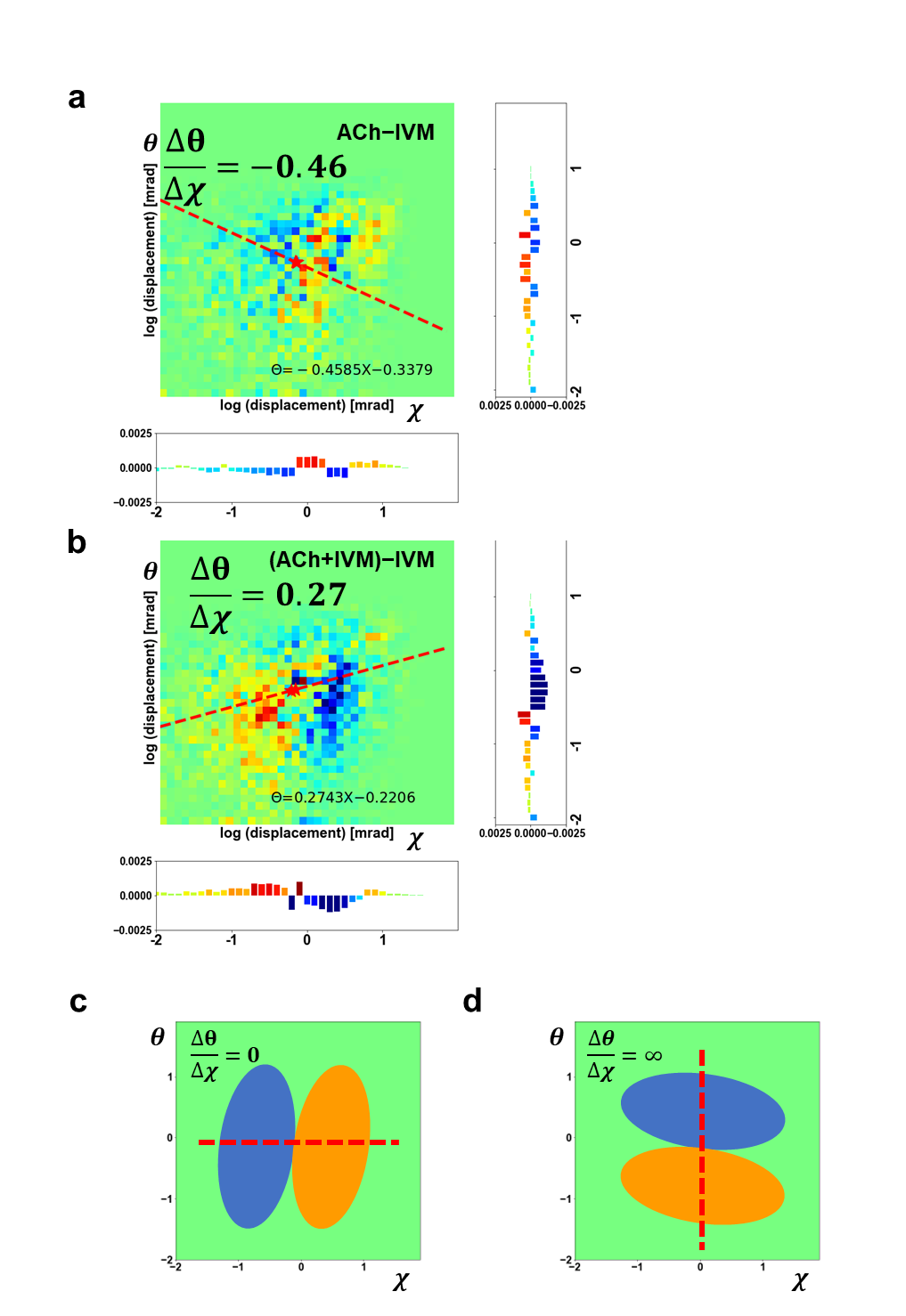
**

Schematic diagram of the difference 2D motion maps which only depends on twisting (**a**) or tilting (**b**) motions. Difference 2D motion maps between ACh and IVM (**c**), and between ACh+IVM and IVM (**d**).

**Fig. S4 Normalized 2D motion maps**


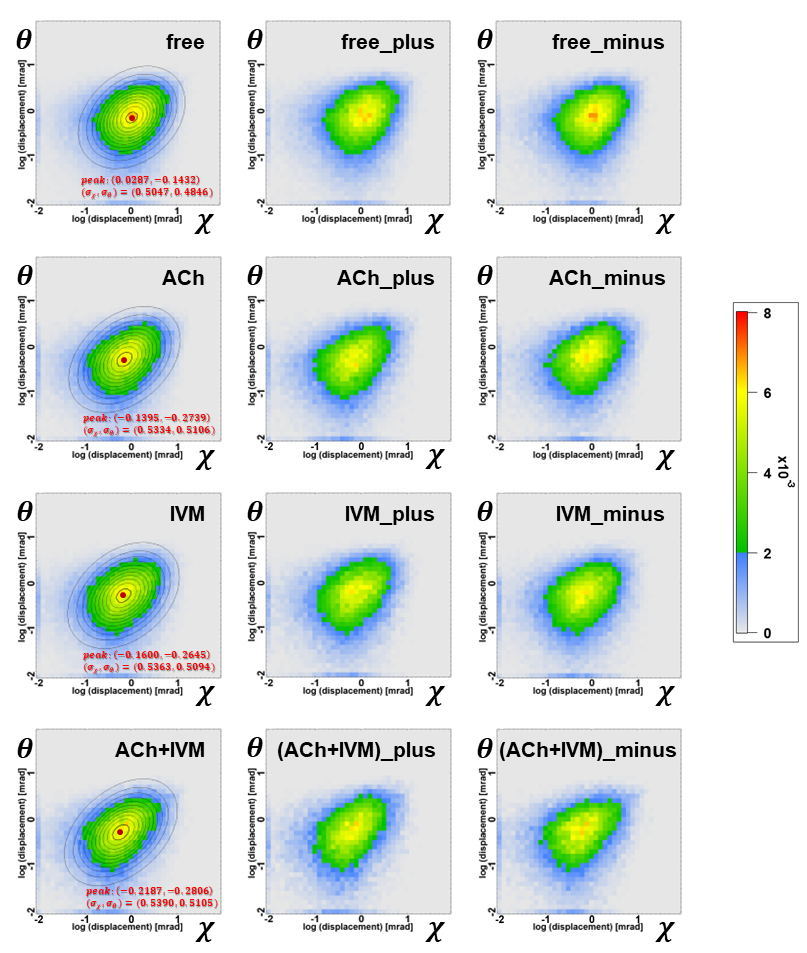


Normalized 2D motion maps of nAChR α7 in normal, positive and negative directions of the 𝝌 axis in all conditions.

**Fig. S5 MSD curves and statistical analysis**


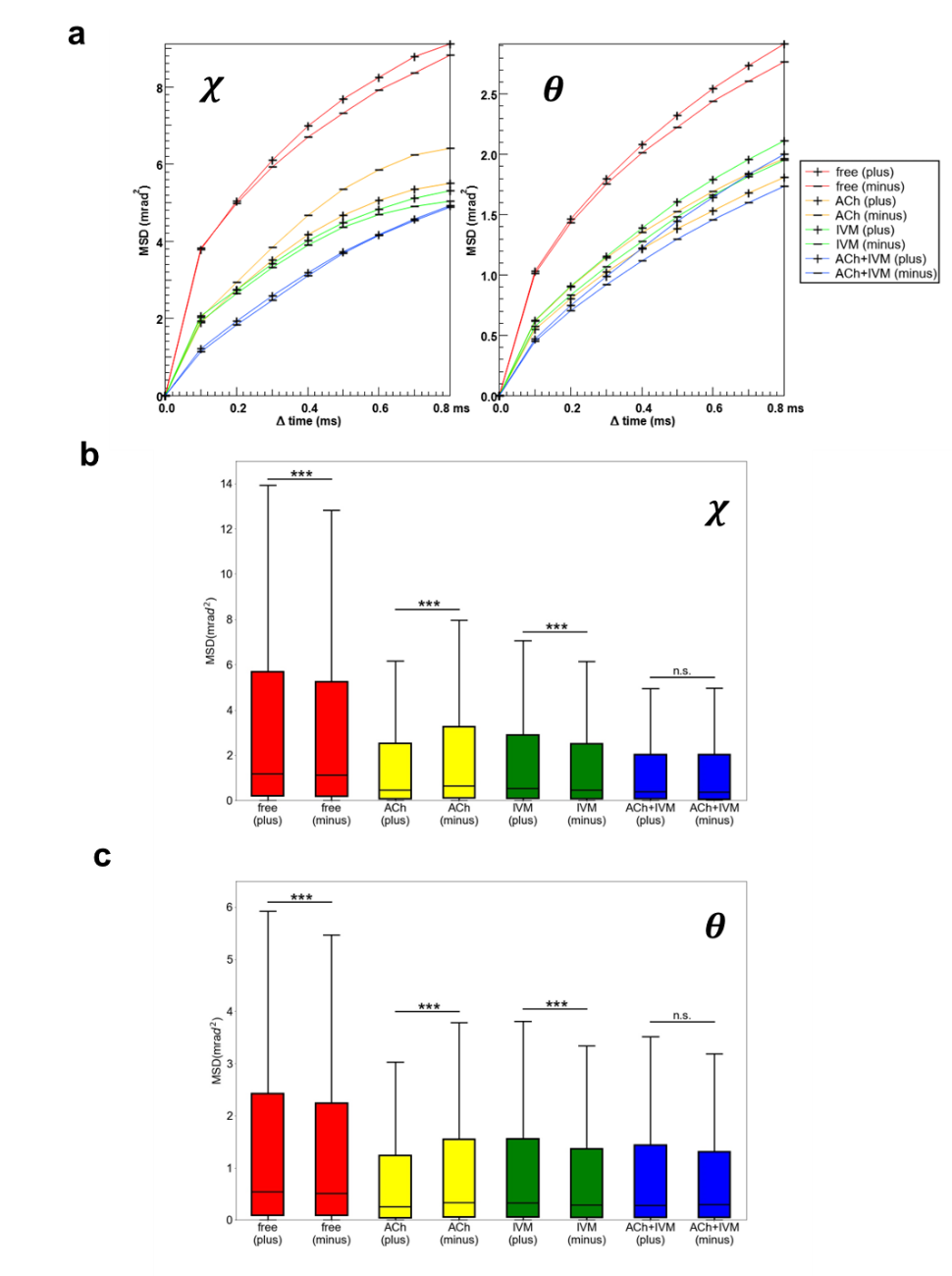


**a** MSD curves of twisting and tilting motion of nAChR α7 in the positive and negative directions of the 𝝌 axis in all conditions. The boxplot analysis (displaying median values of MSD) and Wilcoxon Rank-Sum Test of MSD values for nAChR α7 in the **b** twisting and **c** tilting directions between the positive and negative directions along the 𝝌 axis for all conditions. Asterisk shows that p-value between the positive and negative directions along the 𝝌 axis is statistically significant for free, ACh, and IVM (* p < 0.1, ** p < 0.01, *** p < 0.001).

**Fig. S6 The angular displacement distributions**


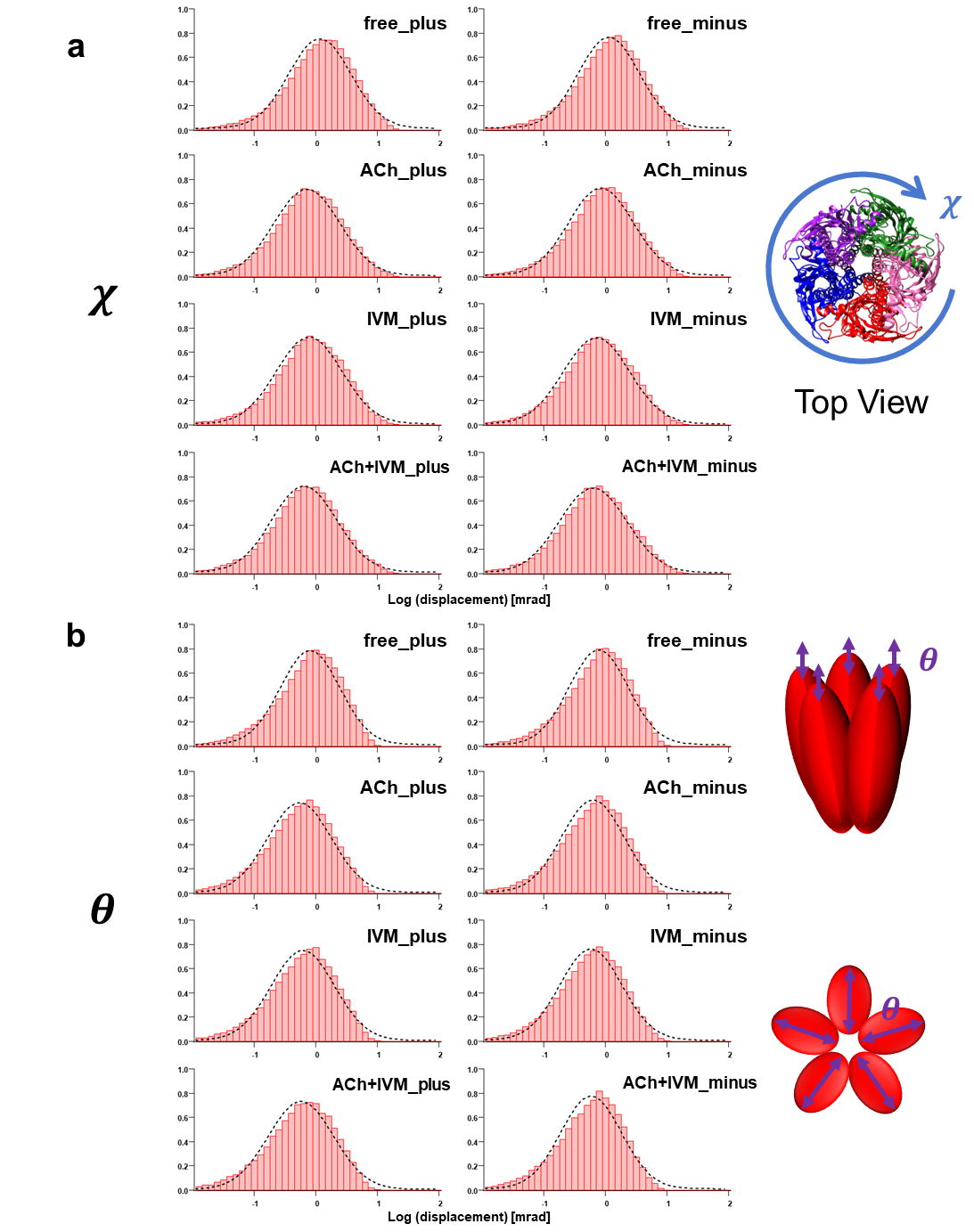


The angular displacement distributions of twisting (**a**) and tilting (**b**) motions of nAChR α7 in the positive and negative directions of the 𝝌 axis in all conditions.

**Fig. S7 Difference angular displacement distributions**
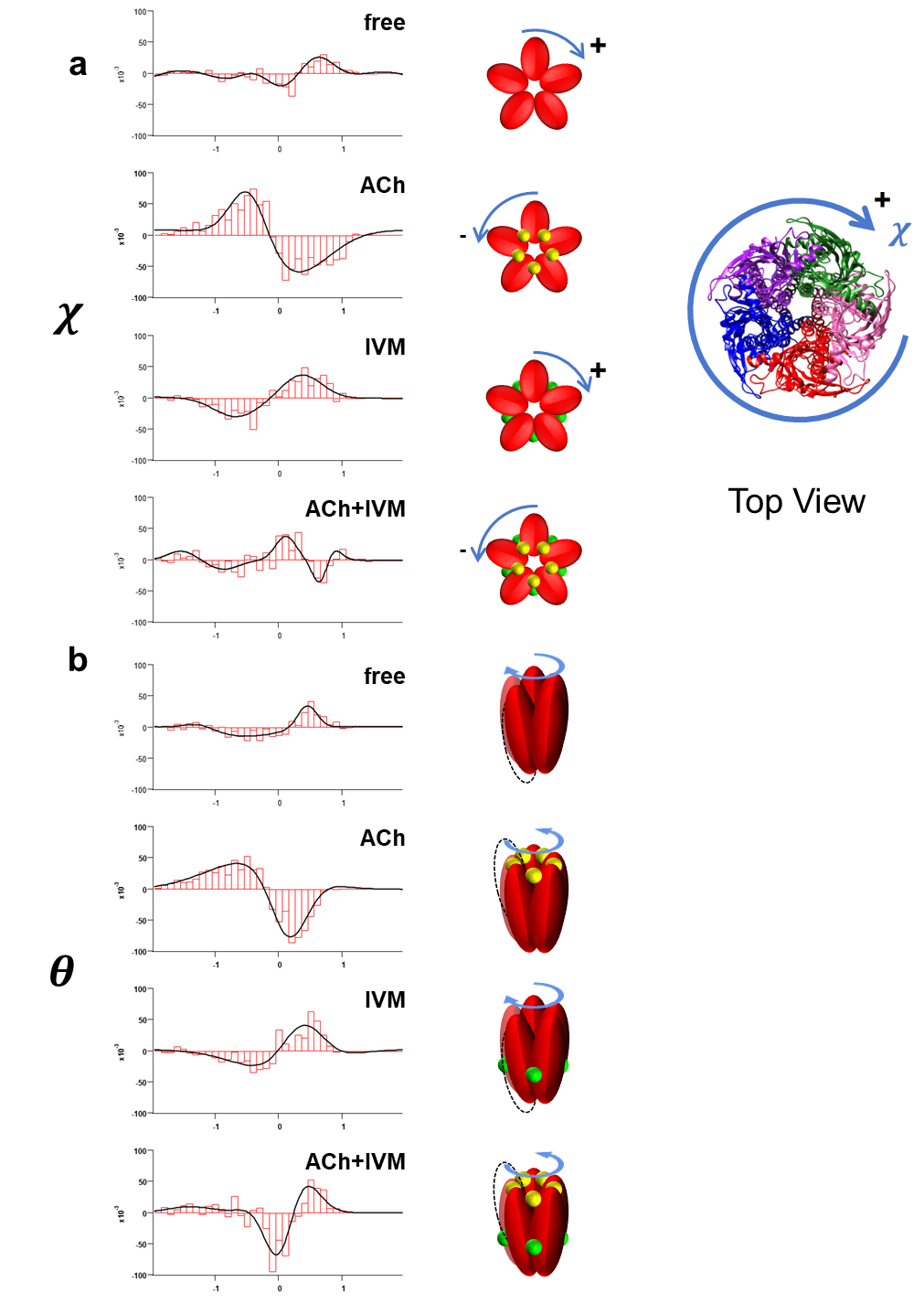


The fitting of difference angular displacement distributions between the positive and negative directions of twisting motion (**a**) and tilting motion (**b**) at this point for all conditions.

**Fig. S8 Difference 2D motion contour map**

**
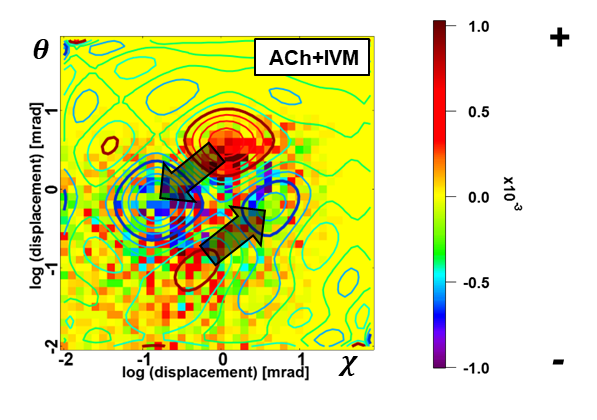
**

The difference 2D motion contour maps between the positive and negative directions in the 𝝌 axis for ACh+IVM condition. The red area is the area of positive 𝝌 motion, and the blue area is the area of negative 𝝌 motion.

**Table 1 Parameters of the DXT experiments**

| Time resolutions | 100 μs |
| --- | --- |
| Wavelength | 0.7~1.5 Å |
| Beam size at the sample position | 50 μm × 50 μm |
| Photon flux | 6×10^14^ photon/sec |
| Photon energy at the sample | 8~17keV |
| Integrated dose | 34 kGy |
| Graft density of the sample | 1 particle/μm^2^ |

The detailed parameters of the DXT experiments.
